# Effect of interval time between hysterosalpingography and intrauterine insemination on the pregnancy outcome of infertile patients

**DOI:** 10.3389/fendo.2023.1175278

**Published:** 2023-10-24

**Authors:** Li Ling, Mengzhu Chen, Tao Shen, Fang Yang, Yihan Jin, Yuanjiao Liang

**Affiliations:** ^1^ Reproductive Medicine Center, Zhongda Hospital, School of Medicine, Southeast University, Nanjing, China; ^2^ Department of Obstetrics and Gynecology, Zhongda Hospital, School of Medicine, Southeast University, Nanjing, China

**Keywords:** hysterosalpingography, intrauterine insemination, infertility, live birth rate, clinical pregnancy

## Abstract

**Background:**

Hysterosalpingography (HSG) is the most commonly applied tubal patency test in clinical practice. Although some studies have found an increased pregnancy rate after HSG, no studies to date have specifically characterized the effect of interval time between HSG and IUI on pregnancy outcome.

**Objectives:**

To investigate the effect of interval time between HSG and intrauterine insemination (IUI) on live birth rates of infertile patients.

**Methods:**

Retrospective cohort study. The reproductive medical record system was used to identify patients who completed ≥1 IUI cycle between January 2017 and October 2021. According to the interval time between HSG and IUI, patients were divided into three groups: <6months interval group,6-12 months interval group and >12 months interval group. The generalized estimating equation with Poisson distribution was used to estimate the risk ratios (RRs) and 95% confidence intervals (CIs) of different groups.

**Results:**

A total of 413 patients completed 701 IUI cycles during the study period, <6months interval group, 415 cycles; 6-12 months interval group, 138 cycles; >12 months interval group, 148 cycles. The live birth rate of <6 months group was higher than other two groups (17.35% vs. 12.32% vs. 8.11%, P=0.017); Similarly, the clinical pregnancy rate of <6 months group was also higher than other two groups (19.76% vs. 14.49% vs.11.49%, P=0.049). When adjusted separately for FSH, AMH, infertility type, duration of infertility, infertility diagnosis, total motile count (TMC) of sperm, medications, endometrium size and dominant follicle size, the live birth rate of >12 months group severally significantly decreased by 60% (adjusted RR = 0.40, 95% CI [0.19–1.40]). The cumulative clinical pregnancy and live birth rates of <6 months group were higher than other two groups (P<0.05), but the cumulative pregnancy rate among three groups were not statistically different (log rank test: P=0.06).

**Conclusion:**

The interval time between hysterosalpingography and IUI is related to pregnancy outcome. The clinical pregnancy and live birth rates were the highest when the time interval was less than 6 months. Therefore, IUI should be recommend as soon as possible after HSG if the patient couple meets the IUI indication.

## 1 Introduction

Intrauterine insemination (IUI) is a frequently utilized method of assisted reproduction that injects the optimized husband’s sperm suspension into the female uterine cavity through non-sexual intercourse to obtain pregnancy (1). IUI is safe and non-invasive, is simple and convenient to operate, and has fewer complications. Patient- and cycle-specific factors affect IUI outcomes, such as the type of infertility, the age of the woman, body mass index (BMI), infertility diagnosis, semen parameters, stimulation regimens, number of follicles before ovulation, and thickness of the endometrium during ovulation (2–4). However, the factors affecting the outcome of IUI have not been fully revealed.

The assessment of tubal patency is necessary before IUI, which requires that at least one side of the fallopian tube shows patency. Hysterosalpingography (HSG) is the most commonly applied tubal patency test in clinical practice in many countries. Interestingly, some studies have found an increased pregnancy rate in the months after HSG (5–7). The mechanism by which HSG can improve pregnancy rates may lie in its flushing effect on the fallopian tubes, which can remove any debris or mucus blocking the free transport of oocytes, sperm, and/or embryos. A considerable number of randomized trials have sought to identify the effect of tubal flushing (8), but the timeliness of its efficacy is unknown.

Hysterosalpingography can increase the chance of pregnancy, but the therapeutic effect of hysterosalpingography is weakened if the fragments accumulated in the fallopian tubes over time due to events such as retrograde menstruation and tubal discharge. The more time that has passed since a tubal flushing event, the less impact is expected of this event. Therefore, it can be guessed that tubal flushing has a certain timeliness, but the effect of the interval time between hysterosalpingography and IUI on pregnancy outcome is largely unknown. Therefore, we retrospectively analyzed the IUI data of a single center to assess the impact of interval time.

## 2 Materials and methods

### 2.1 Study material

This study was a retrospective cohort study of IUI cycles in the Department of Reproductive Medicine Center, Zhongda Hospital, School of Medicine, Southeast University from January 2017 to October 2021. Prior to IUI, both husband and wife underwent routine examination, including fallopian tube patency examination (HSG with a water-based contrast medium), ovulation monitoring under vaginal ultrasound, hormone measurement on the 2nd to the 5th day of the menstrual cycle. The male partner underwent a sperm function test (at least twice), including a period of abstinence, sperm density, sperm count, volume, motility, and normal sperm morphology after abstinence for 2 to 7 days. The inclusive criteria included the following: the patency of at least one tube should have been confirmed by hysterosalpingography and no history of sexually transmitted diseases or lower abdomen/pelvic surgery. The exclusion criteria were also as follows: the HSG time is unknown and the pregnancy outcome was absent. All patients met the indications of IUI treatment and were informed of the IUI treatment, which they agreed to adopt. The non-ovulating patients signed an ovulation induction informed consent form.

This study was approved by the Ethics Committee of Zhongda Hospital, School of Medicine affiliated to Southeast University, with ethics approval number 2021ZDSYLL199-P01.

### 2.2 Clinical protocols

In our center, single intrauterine insemination with the husband’s sperm is used. If patients had normal menstrual cycles, serum hormone and anti-mullerian hormone (AMH) levels were previously monitored for normal ovulation. The IUI procedure was performed in natural cycles. Patients with irregular menstruation, follicular dysplasia or development arrest, or inability to conceive in two natural cycles should be given appropriate stimulation programs according to the situation.

In stimulation cycles, pelvic ultrasound and serum hormone levels [follicle-stimulating hormone (FSH), luteinizing hormone (LH), and estradiol] were evaluated on the 2nd and 4th day of the cycle. Once the patients were confirmed to be in the early follicular phase, letrozole (Jiangsu Hengrui Medicine Co., China), at 2.5 to 5 mg daily for 5 consecutive days from the same day, was added. We performed transvaginal ultrasound from menstrual cycle (MC) day 10 to ensure that a follicle had been recruited. The follicle diameter was measured in two perpendicular planes and was averaged for both planes. If the leading follicle size was less than 12 mm on the 10th day, 75 IU human menopausal gonadotropin (Lizhu Pharmaceutical Trading Co., China) was added depending on the ovarian response. If a leading follicle measured 18 mm or more, the trigger time was selected according to the level of LH. If the blood LH level did not reach more than 2.5 times of the base value, 0.1 mg GnRH-a (decapeptyl, Ferring Pharmaceuticals) or 10,000 IU human chorionic gonadotropin (hCG; Lizhu Pharmaceutical Trading Co., China) would be injected to induce ovulation. IUI was then performed 24–36 h later. If an LH surge (the LH surge was defined as an LH level that reached more than 2.5 times of the base value) was detected, the IUI was performed on the next day.

In nature cycles, pelvic ultrasound and serum hormone levels were also evaluated on the 2nd to the 4th day of the cycle. The patients received continuous pelvic ultrasound and serum hormone level examination every 2 days from MC day 10 to 12. Once a leading follicle measured 18 mm or more, an ovulatory trigger with GnRH-a or hCG was injected. IUI was then performed 24–36 h later. If an LH surge was detected before an ovulatory trigger, the IUI was performed on the next day.

### 2.3 Sperm preparation

IUI sperm samples were collected and prepared according to the 5th edition of the WHO Human Semen Inspection and Treatment Laboratory Manual. After an abstinence period of 2–7 days, sperm was collected by masturbation on the day of IUI. The IUI sperm samples were collected by gradient concentration centrifuging, using the 45%–90% SpermGrad ™ density gradient system (10138, Vitrolife, Västra Frölunda, Sweden). Centrifugation was performed at 400 ×*g* for 20 min. After collection, the sperm was washed once with 3 mL of SpermRinse (10101, Vitrolife, Västra Frölunda, Sweden) for 10 min at 200 ×*g*. The sperm was then suspended in 0.5mL of IVF plus (10136, Vitrolife, Västra Frölunda, Sweden). The post-preparation semen samples were cultured in an incubator at stable conditions of 6% CO_2_ at 37°C until IUI. Prior to performing IUI, sperm density and mobility were assessed. We finally calculated the post-preparation total motile count (TMC) of sperm [TMC = volume (mL) × count (10^6^/mL) ×percent motility].

### 2.4 IUI procedure

For the IUI, only one insemination procedure was performed in each cycle. The patient took the bladder lithotomy position, disinfected the vulva with normal saline, washed the vagina, removed cervical mucus, and dried it with aseptic dry gauze. A soft catheter (Cook Group, Bloomington, Indiana) with 0.5 ml of sperm suspension was entered into the fundus of the uterine cavity and retreated 1 cm, and then the sperm suspension was slowly pushed until it was completely injected. After the procedure, the patients rested on the examination table in the supine position for 20 min.

### 2.5 Luteal phase support method

All patients took dydrogesterone tablets for luteal support on the second day of IUI. Moreover, 10 mg b.i.d. oral dydrogesterone (Duphaston; Abbott Biologicals, Chicago, IL, USA) was administered for 2 weeks after IUI. If pregnancy occurred, the medication was continued until 8 to 10 weeks of gestation. However, luteal support was discontinued if not pregnant.

### 2.6 Pregnancy outcome

Blood was drawn to detect HCG beginning at 2 weeks after the day of IUI. Once pregnancy was confirmed, luteal support was continued. The patients received early pregnancy ultrasound examination at 4 weeks after IUI.

The primary outcome was live birth rate. Secondary outcomes were pregnancy rate and clinical pregnancy rate. Live birth was defined as a live newborn born after 28 weeks of gestation. Pregnancy was defined as a positive serum hCG level 14 days after IUI (>50 mIU/mL). Clinical pregnancy was defined as an intrauterine gestational sac visible on ultrasound at 6 weeks of gestational age. The live birth rate was calculated by dividing the number of live births (twins are a single count) by the number of IUI cycles.

### 2.7 Statistical analysis

At baseline, the patients’ characteristics were described by the interval time groups. The analysis of variance, Wilcoxon rank sum test, or chi-square test were used to evaluate the difference between groups based on the types of the variables. The generalized estimating equation with Poisson distribution was used to estimate the risk ratios (RRs) and 95% confidence intervals (CIs) of different groups. ARR <1 indicated a reduction in pregnancy rate. Some factors known or suspected to be associated with outcomes were used as covariates and were adjusted in the models, including female age, BMI, type of infertility, duration of infertility, reason of infertility, FSH, AMH, TMC, type of medications used for controlled ovarian stimulation, endometrium size, and dominant follicle size. The time when the first IUI was performed was regarded as a starting point for observation, and the cumulative pregnancy rate for different numbers of IUI cycle was calculated using the Kaplan–Meier method. All of these analyses were run using R software, and two-sided probability values of <0.05 were deemed to be statistically significant.

## 3 Results

A total of 413 patients completed 701 IUI cycles during the study period: <6 months interval group, 415 cycles; 6–12 months interval group, 138 cycles; and >12 months interval group, 148 cycles. The baseline characteristics of women including age, BMI, FSH, AMH, TMC, type of infertility, type of medications used for controlled ovarian stimulation, duration of infertility, infertility diagnosis, endometrium size, and dominant follicle size were not statistically different between the three groups (**Table 1**).

**Table 1:** Patient characteristics (recorded at the first clinic visit during the study period).

	<6 months	6–12 months	>12 months	*P*
Number of patients	265	75	73	
Number of cycles	415	138	148	
Age, years: mean (SD)	30.35 (3.97)	30.93 (3.77)	30.33 (3.19)	0.479
Type of infertility				0.458
Primary infertility	171 (65.3)	49 (65.3)	42 (57.5)	
Secondary infertility	91 (34.7)	26 (34.7)	31 (42.5)	
Duration of infertility, years	2.00 [1.00, 3.00]	2.00 [1.00, 3.00]	2.00 [1.00, 3.00]	0.592
Infertility diagnosis, *n* (%)				0.527
Ovulatory	108 (57.1)	25 (52.1)	37 (69.8)	
Endometriosis	7 (3.7)	3 (6.2)	1 (1.9)	
Tubal	43 (22.8)	15 (31.2)	9 (17.0)	
Other	10 (5.3)	2 (4.2)	1 (1.9)	
Unexplained	21 (11.1)	3 (6.2)	5 (9.4)	
BMI, kg/m^2^: mean (SD)	22.66 (3.11)	22.81 (3.16)	23.51 (3.49)	0.135
FSH, median [IQR]	7.20 [6.05, 8.30]	7.12 [5.75, 8.99]	6.99 [5.73, 8.17]	0.625
AMH, median [IQR], ng/mL	4.62 [2.91, 7.32]	5.16 [3.03, 7.55]	4.95 [3.17, 10.62]	0.325
Total motile sperm count × 10^6^	31.75 [21.80, 41.30]	33.20 [24.20, 40.65]	32.20 [20.40, 38.45]	0.402
Medications				0.343
None	36 (13.6)	15 (20.0)	12 (16.4)	
Letrozole	123 (46.4)	29 (38.7)	26 (35.6)	
Letrozole with gonadotropins	106 (40.0)	31 (41.3)	35 (47.9)	
Endometrium size (mm), mean (SD)	10.15 (2.08)	10.09 (2.21)	10.00 (2.22)	0.762
Dominant follicle size (mm), mean (SD)	19.96 (2.34)	19.74 (2.28)	19.99 (2.28)	0.690

The pregnancy outcomes for different groups based on IUI cycles are shown in **Table 2**. The live birth rate of the <6 months group was higher than those of the other two groups (17.35% vs. 12.32% vs. 8.11%, *P* = 0.017); Similarly, the clinical pregnancy rate of the <6 months group was also higher than those of the other two groups (19.76% vs. 14.49% vs. 11.49%, *P* = 0.049). Although the pregnancy rate was not statistically different among different groups (21.21% vs. 15.94 vs. 14.19%, *P* = 0.111), the pregnancy rate had a downward trend, which decreased with an increase of interval time.

**Table 2:** Pregnancy outcome for different groups based on intrauterine insemination cycles.

Outcome	<6 months *N* = 415	6–12 months *N* = 138	>12 months *N* = 148	*χ* ^2^	*P*
Pregnancy	88 (21.21)	22 (15.94)	21 (14.19)	4.38	0.111
Clinical pregnancy	82 (19.76)	20 (14.49)	17 (11.49)	6.05	0.049
Live birth	72 (17.35)	17 (12.32)	12 (8.11)	8.16	0.017

The RRs of pregnancy for different groups based on generalized estimating equation are shown in **Table 3**. Overall, when adjusted separately for female age and BMI or FSH, AMH, infertility type, duration of infertility, infertility diagnosis, TMC, medications, endometrium size, and dominant follicle size, compared with the <6 months interval group, the live birth rate of the >12 months group significantly decreased separately by 50% [adjusted RR = 0.50, 95%CI (0.32–0.75)] and 60% [adjusted RR = 0.40, 95% CI (0.19–1.40)]. Compared with the <6 months interval group, the clinical pregnancy rate of the >12 months group was also statistically different [adjusted RR = 0.51, 95% CI (0.30–0.86)].

**Table 3:** Risk ratios of pregnancy for different groups based on generalized estimating equation with Poisson distribution.

	Crude RR	Adjusted RR^a^	Adjusted RR^b^
Pregnancy			
<6 months	1.0	1.0	1.0
6–12 months	0.75 (0.56, 1.01)	0.83 (0.61, 1.11)	0.84 (0.54, 1.30)
>12 months	0.67 (0.49, 0.91)	0.70 (0.51, 0.95)	0.67(0.41, 1.08)
Clinical pregnancy			
<6 months	1.0	1.0	1.0
6–12 months	0.73 (0.54, 1.00)	0.81 (0.60, 1.11)	0.71 (0.46, 1.11)
>12 months	0.58 (0.41, 0.82)	0.61 (0.43, 0.86)	0.51 (0.30, 0.86)
Live birth			
<6 months	1.0	1.0	1.0
6–12 months	0.71 (0.50, 1.00)	0.79 (0.56, 1.11)	0.90 (0.58, 1.40)
>12 months	0.47 (0.31, 0.71)	0.50 (0.32, 0.75)	0.40 (0.19, 1.40)

a Adjusted for female age and body mass index.

b Additionally adjusted for follicle-stimulating hormone, anti-mullerian hormone, infertility type, duration of infertility, the reason of the infertility, total motile count and medications, endometrium size, and dominant follicle size.

**Figure 1:**
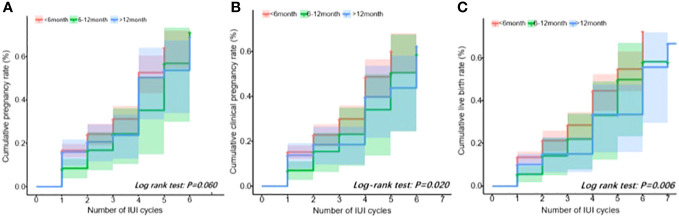
Cumulative outcome rate plot. **(A)** Positive pregnancy test. **(B)** Clinical pregnancy. **(C)** Live birth.

The cumulative pregnancy outcome rate plots are shown in **Figure 1**. The cumulative pregnancy rates among the three groups were not statistically different (log rank test: *P* = 0.06, **Figure 1A**), but the cumulative clinical pregnancy and the live birth rates of the <6 months group were higher than those of the other two groups (*P* all less than 0.05, **Figures 1B, C**).

## 4 Discussion

In this retrospective study, we reported that, for the <6-month-interval group, the live birth and clinical pregnancy rates were significantly higher than those of the other two groups. After several covariates were adjusted, compared with the interval of <6 months, the live birth and clinical pregnancy rates in the >12-month-interval group significantly decreased. Furthermore, when comparing the three interval groups, it was found that the cumulative clinical pregnancy and live birth rates of the <6-month-interval group were the highest.

Factors previously associated with pregnancy rates in IUI cycles include the length of infertility, infertility diagnosis, the woman’s age, semen parameters, fertility medication, and the number of mature follicles (9–11). The interval time between hysterosalpingography and IUI has never been used as an index to predict pregnancy in the IUI cycle in the literature. According to previous studies, some studies showed that tubal patency testing with iodine contrast could flush out debris and remove mucus plugs from fallopian tubes. Two previous research which included women with normal fallopian tubes showed that the pregnancy rates were significant after tubal flushing with contrast media than when the tubes were not tested for patency (12, 13). In our study, when comparing pregnancies at different intervals of HSG, the live rate and the clinical pregnancy rate were the highest within 6 months. This indicates that most of the benefits of HSG are seen within the first 6 months. The more time that has elapsed since HSG, the less benefit from this event would be expected.

This is the first study to evaluate the effect of interval time between hysterosalpingography and IUI on IUI pregnancy outcome. The mechanism by which HSG enhances fertility is not clear. Chronic pelvic inflammatory disease and endometriosis may cause pelvic adhesion, and adhesion around the fallopian tube impairs the oviposition function by mechanically interfering with the anatomical relationship between the distal fallopian tube and the ovary. The HSG operation process and its flushing effect may separate mild fallopian tube adhesion and dredge the fallopian tube to a certain extent (14, 15). Some infertile women successfully became pregnant after receiving HSG, but the HSG results showed that the fallopian tube was obstructed, indicating that the contrast medium of iodine did not penetrate into the fallopian tube or abdominal cavity. The results suggested that the contrast agent mechanically dredged the fallopian tube and uterine cavity, and iodized water stimulated the ciliary activity of the fallopian tube epithelium. On the other hand, the contrast medium might play an immune-regulatory role in the pelvic peritoneum and endometrium, improving the immune microenvironment of the pelvic cavity and uterus to reduce, possibly temporarily, barriers to fertilization and embryo implantation (16). An *in vitro* study showed that iodinated contrast media could inhibit phagocytosis by decreasing the percentage of monocytes phagocyting bacteria (17), which means that the iodine contrast agent can reduce the phagocytosis of mononuclear cells on sperm *in vivo*. As we all know, iodine has anti-inflammatory and disinfection effects, which can reduce inflammatory cytokines, so it may have an inhibiting effect on the spread of microbial infections.

There are two kinds of iodinated contrast agents, namely, water-based contrast and oil-based contrast. All of the patients that were included received HSG with a water-based contrast medium due to the following reasons: Firstly, most radiologists performing tubal patency assessment procedures lacked rich photographic skills and experience of using lipiodol. Secondly, there was insufficient evidence of a difference in clinical pregnancy or live birth rates beyond 6 months of HSG between the use of oil-based contrast and water-based contrast (18). Compared to no intervention, regardless of what contrast agent was used, the probability of natural pregnancy would increase within 24 months after HSG (19). Thirdly, the disadvantage of lipiodol was that an abdominal plain film needed to be taken 24 h after the angiography, and the report was delayed by 1 day. Lastly, the complication rates after HSG with an oil-based contrast medium were reported to be higher compared with water-based contrast (20). If the oil enters the blood vessel in reverse flow, there is a risk of embolism; the impurities in iodized oil sometimes cause allergic reactions. In the past, allergy tests should be carried out before radiography; iodine oil is absorbed slowly and stays in the body for a long time. Occasionally, chronic inflammation of the fallopian tube may occur, and granuloma may form. While water-based contrast has low viscosity, fast flow rate, and fast diffusion, diffusion can be photographed 20 min after operation, and the inspection results can be reported on the same day, which is convenient and fast.

### 4.1 Strengths and limitations

The strength of our study is that the statistical approach which accounts for multiple IUI cycles per couple allowed us, for the first time, to estimate the total effects of interval time between hysterosalpingography and intrauterine insemination on pregnancy outcome. Live birth was the assessed endpoint, which more accurately showed the success rate of IUI.

As a retrospective cohort analysis, this study is not without limitations. We were limited by the use of the reproductive medicine system software, which creates the possibility of confounding bias because of unmeasured confounding factors. Due to its small sample size, we could not further analyze a subgroup of patients, such as unexplained infertility. This observational study cannot confirm whether the association between exposure and outcome is causal, and further clinical randomized controlled trials are needed.

## 5 Conclusion

Our study findings suggest that the short interval time between hysterosalpingography and intrauterine insemination is associated with an increased chance of clinical pregnancy and live birth. The clinical pregnancy and live birth rate were the highest when the time interval was less than 6 months. These findings offer valuable insight to provide patients with anticipatory counseling. We recommend receiving IUI treatment as soon as possible after HSG if the patient couple meets the IUI indication. Further larger studies are needed to validate our result.

## Data availability statement

The original contributions presented in the study are included in the article/**Supplementary Material**. Further inquiries can be directed to the corresponding author.

## Ethics statement

The studies involving human participants were reviewed and approved by the Ethics Committee of Zhongda Hospital Affiliated to Southeast University. The patients/participants provided their written informed consent to participate in this study.

## Author contributions

Material preparation, data collection and analysis were performed by LL, MC, TS, FY, and YJ. YL appraised and revised the manuscript. All authors contributed to the article and approved the submitted version.
